# A bottom-up characterization of transfer functions for synthetic biology designs: lessons from enzymology

**DOI:** 10.1093/nar/gku964

**Published:** 2014-11-17

**Authors:** Max Carbonell-Ballestero, Salva Duran-Nebreda, Raúl Montañez, Ricard Solé, Javier Macía, Carlos Rodríguez-Caso

**Affiliations:** 1 ICREA-Complex Systems Laboratory, Universitat Pompeu Fabra, 08003 Barcelona, Spain; 2 Institut de Biologia Evolutiva, CSIC-UPF, Psg. de la Barceloneta 37, 08003 Barcelona, Spain; 3 Santa Fe Institute, 1399 Hyde Park Road, Santa Fe, NM 87501, USA

## Abstract

Within the field of synthetic biology, a rational design of genetic parts should include a causal understanding of their input-output responses—the so-called transfer function—and how to tune them. However, a commonly adopted strategy is to fit data to Hill-shaped curves without considering the underlying molecular mechanisms. Here we provide a novel mathematical formalization that allows prediction of the global behavior of a synthetic device by considering the actual information from the involved biological parts. This is achieved by adopting an enzymology-like framework, where transfer functions are described in terms of their input affinity constant and maximal response. As a proof of concept, we characterize a set of Lux homoserine-lactone-inducible genetic devices with different levels of Lux receptor and signal molecule. Our model fits the experimental results and predicts the impact of the receptor's ribosome-binding site strength, as a tunable parameter that affects gene expression. The evolutionary implications are outlined.

## INTRODUCTION

The advance of genetic engineering has made it possible to modify genetic programs inside cells by re-designing them in predefined ways (1). Synthetic biology has emerged as a discipline in which modular biological parts are used for the construction of genetic devices. As in any engineering discipline, mathematical and computational models provide the workbench to infer system-level behavior from the properties of the biological parts (2). Standard engineering predicts output responses of a device given a set of input signals and a specified internal set of pieces. Within synthetic biology, the proper characterization of simple blocks in a reliable way constitutes a major challenge for the building of complex genetic devices (3–5).

The transfer function, a term borrowed from electronics, is the representation of the relationship between the input and the output of a system (6,7). This concept has been translated within synthetic biology as the response of a regulable genetic device in the presence of a signal that acts as the control variable of the system. In most relevant scenarios, nonlinear responses are often desirable in order to implement the digital logic abstraction found in man-made circuits. This can be achieved using mechanisms such as saturation of biochemical systems (8), ultrasensitivity (9), multistability (10) and transcription factor cascades (11) among others. Hill functions have been commonly used for the fitting of experimental datasets in biochemistry (12), computational biology, (13), pharmacology (14), systems and synthetic biology (10,15–18). The success of this approach comes from the fact that fitting data require little *a priori* knowledge of the underlying biological mechanisms, and provide quantitative information about affinity and cooperativity of the system (8).

In genetics, Hill-like functions come from the assumption of cooperative effects due to transcription factor multimerization (19) and can be derived from equilibrium calculations on ligand-receptor binding. However, in most cases, its representation results from the correction of the hyperbolic Michaelis–Menten approach by adding an empirical exponent *n* (14), written as

(1) \begin{eqnarray*} \frac{v}{V_{\rm m}{\rm }}={[{\rm S}]^n \over {\rm S}{\rm }^n_{0.5} + [{\rm S}]^n}. \end{eqnarray*}

As a consequence of its empirical nature, neither the original Michaelis–Menten premises nor biological information remains in the model, losing the link between the kinetic parameters and biological mechanisms. Accordingly, models constructed by fitting have very limited predictive value beyond the exact conditions in which data were acquired. Thus, the approximation taken is largely a heuristic one.

Design often requires iterative optimization steps. However, any device modification may lead to some type of unpredictable behavior, forcing further empirical characterization. Unfortunately, such a circumstance is not rare in the process of construction and testing of a genetic device (4). Hence, there is a need for a more suitable framework that allows predictions and avoids time-consuming data collection.

In this regard, the Michaelis–Menten approach (20) may offer an inspiring alternative to the broadly accepted Hill fitting. Interestingly, the transfer function concept fairly matches the substrate-velocity plot for enzymatic catalysis. This classical plot constitutes a clever characterization of enzyme kinetics, connecting a simple experimental setup with a biochemically grounded model, based on very precise premises. In that way, an analogous perspective for genetic devices would confer to transfer functions a desirable predictive value. The aim of this work is to establish a quantitative relation between input's affinity, signal amplitude and the variation of the control variable (i.e. induction molecules). In order to provide an experimental validation, we shall compare our model predictions with the characterization of an engineered device: the Lux system.

The quorum sensing Lux system has been extensively used in synthetic biology (15,16,21). With a sophisticated regulation in nature (22), its engineered versions have been restricted to the transcriptional level, to which a Hill-like behavior with a wide range of cooperativities has been reported (7,13,16,23).

**Figure 1.**
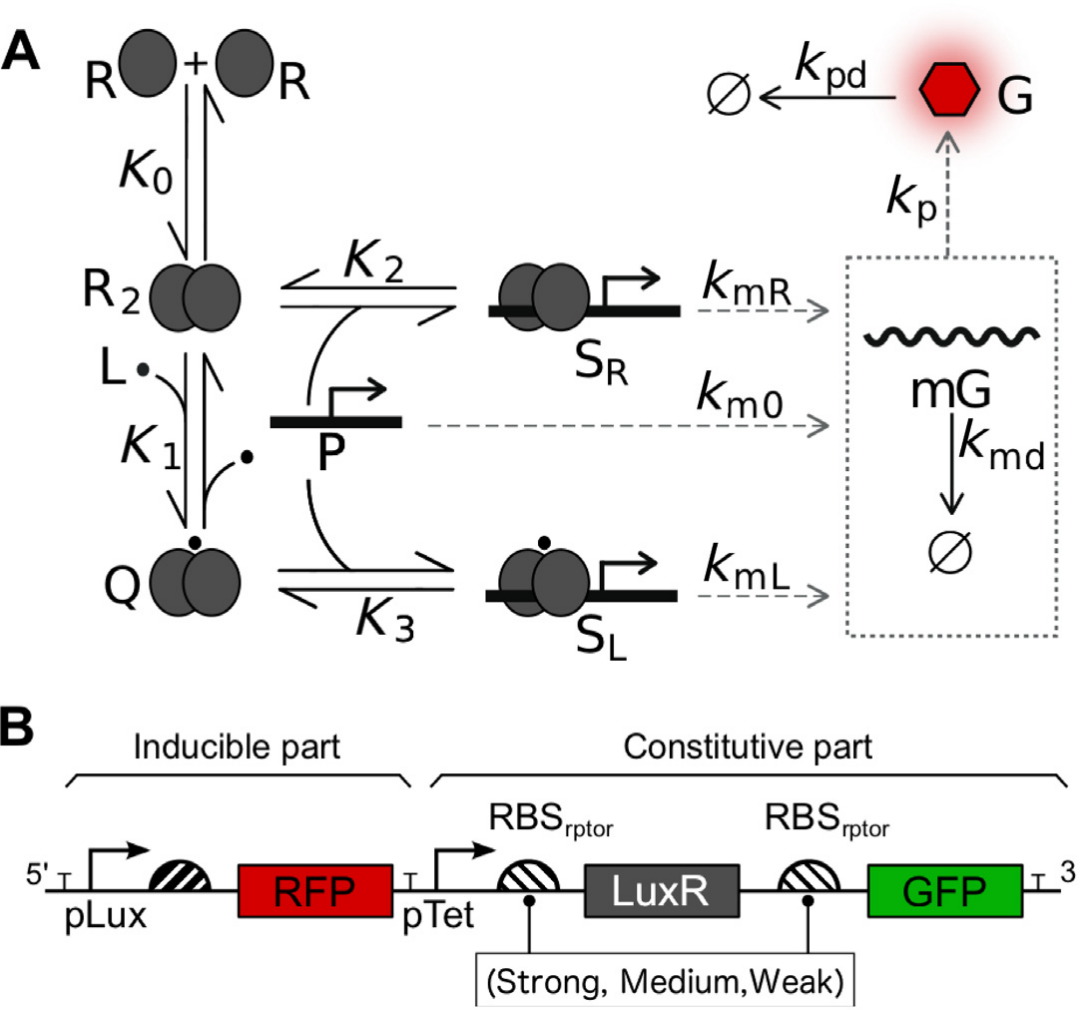
Schematic representation of genetic regulation for the inducible LuxR-pLux engineered devices used in this study. Notation follows the mathematical model: R LuxR receptor, L lactone, and P pLux promoter, R_2_ dimerized receptor without lactone, Q dimerized receptor with lactone, S_R_ transcriptional complex not mediated by lactone, S_L_ lactone mediated transcriptional complex, mG mRNA of reporter gene and G reporter protein. The *K*_*i*_ notation represents equilibrium constants, while *k*_*i*_ refers to kinetic constants (**A**). Genetic architecture of the three constructs analyzed (**B**).

When we look at the biochemical characterization, the interaction of LuxR dimer with 3-oxo-*C*_6_-homoserine lactone (3OC6HSL) induces the binding to promoter (24). This process is mediated in a noncooperative manner by 3OC6HSL, as suggested by studies in Lux and its Car homolog systems (25,26). Interestingly, receptor without lactone cognate is able to bind the DNA promoter (27), suggesting that some expression mediated by free receptor may occur. This scenario, schematically represented in Figure 1A, provides a starting point for a more biologically meaningful model of this system. However, one issue remains: the ability to control and manipulate the elements of the device.

From an engineering perspective, modularity and orthogonal function of genetic parts is the key for the construction of tailored devices. At this point, the ribosome-binding sites (RBSs) are useful elements to control the efficiency of the translation of the mRNA pool. Efforts on the characterization of RBSs variants for different organisms have provided valuable information for the choice of one or another RBS in a genetic system (28). A comparison of the effect of these parts in the expression of the final output is given by its relative strength, which is calculated using a standard value of expression as a reference for normalization (29). The use of different RBSs constitutes a common way to modulate the expression of a particular gene. But what is the impact of RBS changes on the behavior of a device?

To tackle this question, our work presents an enzymology-like approximation that allows us to explore the role of different RBSs in a genetic pLux-LuxR-inducible circuit. This study shows how tunable parts of the device, such as the expression of receptor, modulate the transfer function in a completely predictable manner. As a proof of concept, an experimental characterization and a further mathematical modeling of an inducible genetic device is presented. This picture more close to the biological mechanism suggests some limitations of the convencional Hill fitting approach.

## MATERIALS AND METHODS

### Bacterial strains and growth conditions

Cloning and expression experiments were performed in *Escherichia coli* Top10 (Invitrogen, USA). Cells were grown in Lysogeny Broth (LB) at 37°C and selected with appropriate antibiotics (chloramphenicol 340 μg/ml; kanamycin 250 μg/ml; or ampicilin 100 μg/ml; Sigma, USA). Bacterial strains were preserved in LB glycerol 20% (v/v) at −80°C.

### Construction of 3OC6HSL Lux genetic devices

Cloning was carried out using the Biobrick assembly method and the parts from the Spring 2010 iGEM distribution. The biobrick parts used in this study were the following: *B0014* (double terminator), *B0034* (strong RBS), *B0032* (medium RBS), *B0033* (weak RBS), *R0040* (tetracycline promoter, pTet, as a constitutive promoter), *R0062* (Lux promoter, pLux), *C0062* (LuxR coding sequence), *E1010* (red fluorescence protein, RFP), *E0040* (green fluorescence protein, GFP). Biobrick cloning was performed using an assembly kit (Ginkgo Bioworks, USA).

Three genetic devices were built, composed of a common 3OC6HSL inducible part followed by three variants of a constitutive part, as illustrated in Figure 1B. On one hand, the inducible construct had the following structure: B0014-R0062-B0032-E1010; on the other hand, there were three variants of the constitutive part, cloned as B0014-R0040-X-C0062-X-E0040-B0014, where X corresponds to the three aforementioned alternative variants: *B0034*, *B0032* and *B0033* (labeled as *strong*, *medium* and *weak*, respectively). Technical details about the relative strength of these RBSs are taken from http://parts.igem.org/Ribosome_Binding_Sites/Prokaryotic/Constitutive/Community_Collection. As detailed in the part registry, the B0034 part was used as a standard for normalization. Therefore the relative strengths used in this work were 1, 0.3 and 0.03 for B0034, B0032 and B0033, respectively. All constructs were included in the Biobricks high copy number plasmid (pSB1AK3) and transformed by chemical method. In the case of the experiments with no receptor (see Figures 3C and 5B), a construct bearing only the inducible part within the same plasmid was used. All genetic constructs were confirmed by Sanger sequencing.

### Fluorescence assays for gene expression determination

Strains containing the plasmid of interest were grown overnight in LB ampicillin at 37°C and continuous shaking. A 1000-fold dilution from overnight culture was grown until exponential phase, OD_660_ ≈ 0.4. Cultures were centrifuged at 4000 g, during 5 min and resuspended in fresh LB ampicillin up to an OD_660_ = 0.3. Incubation for *in vivo* measures was carried out by transferring 100 μl of the diluted cultures and 100 μl of LB ampicillin with the appropriate 3OC6HSL (N-[β-ketocaproyl]-L-homoserine lactone; Cayman Chemical Company, USA) concentrations into a flat bottom 96-well microplate (Nunc, Thermo Fisher Scientific, USA). LB without cells was included in the incubation as a background control for both fluorescence and absorbance.

Gene expression was monitored in time for a battery of 3OC6HSL concentrations by quantification of the RFP. LuxR was indirectly reported by measuring the concomitant expression of GFP placed in tandem with LuxR (Figure 1B). Incubation and measures of bacterial cultures during characterization were performed on a Synergy MX microplate reader (BioTek Instruments, USA) every 10 min for 14 h. Fluorescence measures for RFP (ex: 578 ± 9 nm, em: 616 ± 9 nm) and GFP (ex: 478 ± 9 nm, em: 516 ± 9 nm) with gain 70 were carried out, as well as optical density (OD at 660 nm) measures. Incubation was done at 37°C with continuous orbital shaking (medium intensity). 3OC6HSL concentration conditions were prepared from an initial stock at 10^−2^ M (3:1, phosphate buffered saline:ethanol). Serial dilutions in LB ampicillin ranging from 10^−4^ to 10^−10^ M were prepared the day of the experiment.

### Data transformation and Hill function fitting

Sample absorbance and fluorescence readings (OD_660_(S), *f*(S)) were corrected using signal background control (OD_660_(B), *f*(B)). Averaged data were obtained from six independent experiments. As described in (18), output signal *Θ*_*i*_ was calculated according to the formula:

(2) \begin{equation*} \it \Theta _i={f_i({\rm S})-f_i({\rm B}) \over {\rm OD}_{{\rm 660}}({\rm S})- {\rm OD}_{{\rm 660}}({\rm B})} \end{equation*}

where *i* refers to GFP or RFP. The value *Θ* corresponds, with a factor of proportionality, to the concentration of the fluorescent protein *i* per cell. Matlab R2013a software was used for fitting according to the following formula, assuming a cooperative behavior and signal basality:

(3) \begin{equation*} \it \Theta _{{\rm RFP}}=a+ {b[{\rm 3OC6HSL}]^n \over K_{{\rm 0.5}}^n + [{\rm 3OC6HSL}]^n} \end{equation*}

Nonlinear least squares were computed using the trust region algorithm with default settings.

### Time series and signal variation computation

A normalized value of *Θ*_RFP_, *Θ*_RFP_(*norm*), was calculated for every time step as following: given a time, *Θ*_RFP_ for each [3OC6HSL] was divided by the maximal value. We defined signal variation, $\cal S$, as the coefficient of variation of *Θ*_RFP_(*norm*) in a time interval. Evolution of $\cal S$ over time was calculated using a moving window, consisting of five points of *Θ*_RFP_(*norm*), therefore capturing the information of 50 min in total.

The relation between the values of $\cal S$ for the different [3OC6HSL] was evaluated using the estimator *α*. This value, defined as the standard deviation of the different $\cal S$ curves, was used to establish the region of the steady-state condition for transfer function acquisition. According to *α* analysis, the time for the transfer function acquisition was set at 14 h, applying a gain 75 for fluorescence measures.

## RESULTS

### Transfer function acquisition

**Figure 2.**
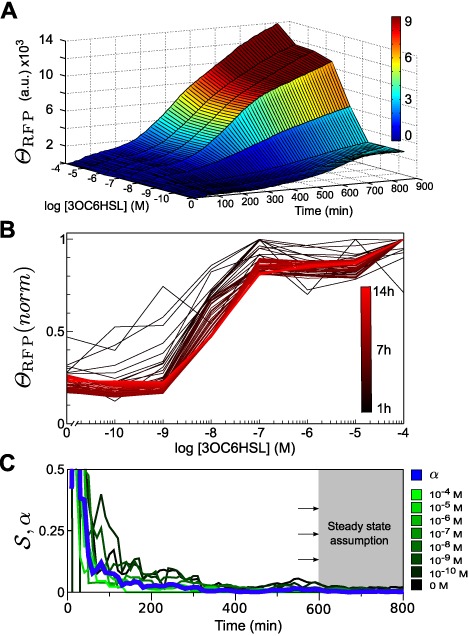
Transfer function acquisition RBS_rptor_ (medium) construct. A 3D chart showing response measurements of RFP expression along time at different [3OC6HSL] (**A**). Time series of normalized RFP expression. Colors (from black to red) indicate different times in the experiment, from 1 to 14 h (**B**). Estimation of the time regime for transfer function acquisition (**C**). In a green scale, the value of $\cal S$ for the eight different [3OC6HSL]. In blue, *α* value accounting for the overall signal variation along time. The shaded area shows the time region in which we assume the steady state has been reached.

The exact time for transfer function acquisition is often arbitrary and still constitutes an open issue (7,30). As an illustrative case, Figure 2A shows the evolution of *Θ*_RFP_ along time for different [3OC6HSL] for the construct with the RBS_rptor_ (medium). Similar qualitative behavior was observed in the other two constructs (data not shown). Representation of *Θ*_RFP_(*norm*) of the different [3OC6HSL] curves allowed a qualitative comparison of the transfer function along time. Figure 2B shows how the transfer function converged (from black to red lines) to a more and more signal overlap.

After the introduction of 3OC6HSL, cells require time for protein production and maturation. At the steady state, i.e. Δ*Θ*/Δ*t* ∼ 0, production is compensated by degradation processes, giving rise to a constant value of *Θ* along time. However, given any arbitrary interval time Δ*t*, Δ*Θ* consists of the genetic behavior due to the actual change associated to protein production and the noise associated to the measure in that interval. This imposes a limitation for the application of the steady state definition.

To overcome this limitation, we used ${\cal S}$ as a way to establish a practical definition for the steady-state condition from experimental data (see Materials and Methods section for definition). As at the steady state biological signal does not contribute to the variation of the output, this one must be given only by noise. In Figure 2C, where the time evolution of $\cal S$ for every [3OC6HSL] (labeled from black to green) is shown, one can see how dispersion values depended on time but also on [3OC6HSL] (inversely proportional). The differences among ${\cal S}$ curves were captured in the time evolution of *α* value (see Materials and Methods section), represented by a blue thick line in Figure 2C. Looking at *α*, we could arbitrarily select a threshold to define the time when the steady steady is assumed for the transfer function acquisition. This mathematical transformation allowed us to usefully collapse the information about the level of fluctuations in a single curve. In our study, we chose a final point at 14 h to perform the transfer function characterization and further modeling fitting.

### Characterization of the pLux device varying RBS_rptor_

**Figure 3.**
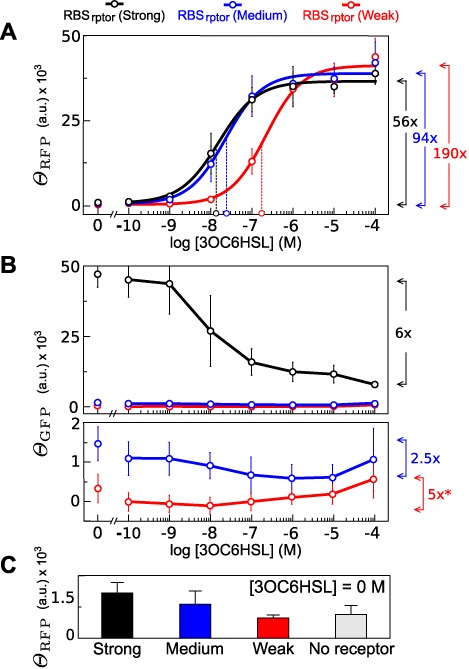
Transfer function varying RBS at receptor. Open circles in the *X*-axis indicate the ${K_{0.5}^n}$ according to the fitting. Arrows in the right side of the chart show the induction with respect to basality according to fitted equations (**A**). Effect of [3OC6HSL] on *Θ*_GFP_, as an estimator of receptor expression. The bottom chart is a zoom of medium and weak constructs. Arrows in the right side of the charts indicate the variation between minimum and maximum value of *Θ*_GFP_. Asterisk indicates that *Θ*_GFP_ was very close to zero giving rise to negative values. In this case, variation was calculated using the lowest positive value (**B**). Leakiness of constructs at [3OC6HSL] = 0 M (**C**).

In order to modulate the strength of gene expression, we characterized a set of constructions using different RBS_rptor_ as illustrated in Figure 1B. The characterization of the respective transfer functions is summarized in Figure 3. The amount of output in response to [3OC6HSL] suggested a noncooperative effect, i.e. *n* ≈ 1 (see Figure 3A and Table 1 for numerical details). Furthermore, the results showed a decrease of the turning point when stronger RBS_rptor_ were used.

**Table 1.** Data fitting for the parameters using Hill function approach and the enzymology-like model assuming leakiness

$\rm {RBS_{rptor}}$	*a*	*b* (× 10^4^)	*K*_0.5_ (× 10^−8^) M	*n*	*r*^2^	*a*_1_ (× 10^4^)	*a*_2_ (× 10^−10^) M	*a*_3_ (× 10^−8^) M	*r*^2^
Strong	638 ± 1990	3.6 ± 0.3	1.5 ± 8.9	0.93 ± 0.16	0.96	3.6 ± 0.1	3.2	1.5± 0.7	0.95
Medium	404 ± 2103	3.8 ± 0.3	2.3 ± 4.8	0.97 ± 0.13	0.95	4.0 ± 0.2	4.0	2.6 ± 1.1	0.95
Weak	215 ± 1605	4.1 ± 0.3	22 ± 59	0.99 ± 0.23	0.96	4.1 ± 0.2	12.7	22.0 ± 6.8	0.96

The expression of receptor monitored by fluorescence measurements of the GFP tandem construction (Figure 1B) showed how the receptor expression was practically unaffected in RBS_rptor_ (medium) and RBS_rptor_ (weak) constructs. However, a significant decrease of *Θ*_GFP_ was observed in the case of the RBS_rptor_ (strong) construct.

Measures with zero ligand concentration produced a basal signal. Such a response was also dependent on the strength of the receptor, as shown in Figure 3C. The analysis of constructs without receptor also produced a basal expression, independently of lactone concentration. This suggests that the engineered pLux alone was able to promote the expression without receptor.

### Enzymology-based model premises

The mathematical model for the inducible device was established according to the following premises:

- Transfer function is evaluated at the steady state where a single stable fixed point is expected.
- Concentrations of molecular species regarding the genetic device, i.e. receptor, promoters and transcriptional and translational machinery, remain invariable along time.
- Total promoter concentration [P_T_] is much smaller than the total transcriptional activator receptor [R_T_].
- Ligand concentration [L] (the input) is assumed invariant in time and, according to the previous premise, large enough to consider that the bound part is negligible with respect to the total one, i.e. [L] ≈ [L_T_].
- Genetic devices work under the condition in which its load neither affects the behavior of the cell nor causes a limitation of resources. Metabolic burden is therefore not included in the formalization.
- The parts of the genetics device have an orthogonal functionality, i.e. the components perform one predefined function and they have not got undesirable interactions with the other cellular components. Therefore, they perform the expected behavior.

Besides these general assumptions, for our specific LuxR device and according to the results shown in the previous section, we shall consider the biological mechanism shown in Figure 1A. In agreement to this, RFP expression is modulated by lactone concentration and both luxR-independent and luxR-dependent leakiness are also considered. In order to provide a more understandable view of this characterization, we formulate a mathematical model using the former set of enzimology-inspired premises.

### A simple model predicts the role of the RBS_rptor_ strength in output amplitude and turning point

Departing from the genetic circuit illustrated in Figure 1A, our model considers a process of sensing a molecule L by joining with a dimeric receptor R_2_. It is worth mentioning that the requirement of more than one L molecule would introduce a power over [L]. For the sake of simplicity, we shall assume that one ligand molecule is enough for receptor's activation and further addition has no impact on the behavior of active receptor complex. This assumption is supported by the noncooperative process observed in our experimental characterization and by the reported evidence previously mentioned in the Introduction section.

In this simplified version, we assume that leakiness due to receptor binding and naked promoter does not occur, i.e. *k*_m0_ = 0 and *K*_2_ = 0. The reactions considered in this simple scenario are:

(4) \begin{eqnarray*} {\rm R} + {\rm R} \rightleftarrows {\rm R}_2 &\Longrightarrow & K_0 = [{\rm R}_2]/[{\rm R}]^2 \end{eqnarray*}

(5) \begin{eqnarray*} {\rm L} + {\rm R}_2 \rightleftarrows {\rm Q} &\Longrightarrow & K_1 = [{\rm Q}]/([{\rm R}_2] [{\rm L}]) \end{eqnarray*}

(6) \begin{eqnarray*} {\rm Q} + {\rm P} \rightleftarrows {\rm S}_{\rm L} &\Longrightarrow &K_3= [{\rm S}_{\rm L}]/([{\rm Q}{\rm }] [{\rm P}]) , \end{eqnarray*}

giving rise to the following differential equations:

(7) \begin{eqnarray*} {{\rm d}[{\rm m}{\rm }{\rm G}] \over {\rm d}t}&=& k_{{\rm m}{\rm L}{\rm }}[{\rm S}_{\rm L}] -k_{{\rm md}}[{\rm m}{\rm G}{\rm }] \end{eqnarray*}

(8) \begin{eqnarray*} {{\rm d}{\rm }[{\rm G}] \over {\rm d}t}&=& k_{{\rm p}}[{\rm m}{\rm G}] -k_{{\rm pd}}[{\rm G}{\rm }]. \end{eqnarray*}

At the steady state, d[mG]/d*t* = d[G]/d*t* = 0, [G*] can be written as a function of the active complex [S_L_]:

(9) \begin{eqnarray*} [{\rm G}{\rm }^*]=\frac{k_{\rm p} k_{{\rm mL}}}{k_{{\rm pd}{\rm }} k_{{\rm md}}} [{\rm S}_{\rm L}]. \end{eqnarray*}

Now we need to obtain a mathematical expression of [G*] in terms of the control variables of this model, i.e. [L], [R_T_] and [P_T_]. These correspond to [3OC6HSL] concentration, externally fixed in the experimental setup; amount of LuxR, kept invariant by its constitutive expression ([R_T_]); and the number of Lux promoters ([P_T_]), defined by a constant population of plasmids.

In this simple model, [R_T_] is a function of [R_2T_], as detailed in the mathematical appendix. For the sake of simplicity and according to the biology of the system, we assume that the process of dimerization is prior to the binding with the ligand. Furthermore, we mathematically impose that the ligand interaction has a negligible effect on the equilibrium between dimer and monomer. According to this, we define two conservation equations:

(10) \begin{eqnarray*} &[{\rm R}_{{\rm 2T}}] =& [{\rm R}_2] + [{\rm Q}] + [{\rm S}_{\rm L}] \end{eqnarray*}

(11) \begin{eqnarray*} &[{\rm P}_{\rm T}] = & [{\rm P}]+ [{\rm Q}] + [{\rm S}_{\rm L}], \end{eqnarray*}

where by the assumption of [R_T_] ≫ [P_T_], [S_L_] is not considered in (10). Applying the equilibrium constant definitions (5) and (6), we rewrite the [S_L_] expression and the conservation Equations (10) and (11) as

(12) \begin{eqnarray*} &[{\rm S}_{\rm L}]= &K_1 K_3 [{\rm P}] [{\rm R}_2] [{\rm L}] \end{eqnarray*}

(13) \begin{eqnarray*} &[{\rm R}_{{\rm 2T}}]=&[{\rm R}_2]( 1+ K_1[{\rm L}]) \end{eqnarray*}

(14) \begin{eqnarray*} &[{\rm P}_{\rm T}]=&[{\rm P}](1+K_1K_3{\rm R}_2[{\rm L}]). \end{eqnarray*}

According to these equations, we can rewrite [S_L_] in function [R_2T_] and [P_T_] in Equation (9), giving rise to the expression

(15) \begin{equation*} [{\rm G}^*]=\frac{\gamma g k_{{\rm mL}} [{\rm P}_{\rm T}] [{\rm R}_{{\rm 2T}{\rm }}]}{K^{-1}_3 + [{\rm R}_{{\rm 2T}}]}\left(\frac{[{\rm L}]}{\left( \frac{K^{-1}_1}{1 + K_3 [{\rm R}_{{\rm {\rm 2T}}}]}\right) + [{\rm L}]}\right), \end{equation*}

where *γ* and *g* are groups of kinetics constants as detailed in Equations (35) and (36) of the mathematical appendix. [R_2T_] can also be written as a function of the normalized RBS strength (*ϕ*), with respect to the canonical RBS part (see the mathematical appendix for further details). Now, by defining

(16) \begin{eqnarray*} &{\rm G}^{{\rm app}}_{\rm m}{\rm } = &\gamma g k_{{\rm mL}}[{\rm P}_{\rm T}] \frac{\phi }{(K_3 r^{\prime })^{-1} + \phi } \end{eqnarray*}

(17) \begin{eqnarray*} &K^{{\rm app}} = &K_1^{-1}\frac{(K_3r^{\prime })^{-1}}{(K_3r^{\prime })^{-1} + \phi }, \end{eqnarray*}

we obtain a more compact expression in a familiar Michaelis–Menten form:

(18) \begin{equation*} [{\rm G}{\rm }^*]= {\rm G}^{{\rm app}}_{\rm m} \frac{[{\rm L}]}{ K^{{\rm app}}+ [{\rm L}]}. \end{equation*}

**Figure 4.**
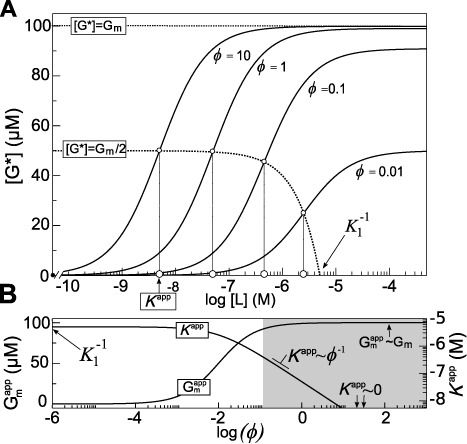
Transfer function for four different *ϕ* according to the simplified model without leakiness. Upper dotted line corresponds to the maximum achievable expression. Lower dotted line shows the ${\rm G}{\rm }^{{\rm app}}_{\rm m}/2$ as function of *ϕ*. The intersections with the transfer functions (little open circles) highlight the ${\rm G}^{{\rm app}{\rm }}_{\rm m}/2$ for the four transfer functions. Their projections over the [L] axis show their respective *K*^app^ values (bigger open circles) (**A**). Dependence of *K*^app^ and ${\rm G}^{{\rm app}}_{\rm m}$ with respect to the value of *ϕ*. Note that when *ϕ* ∼ 0, $K^{{\rm app}}\sim K_1^{-1}$ and ${\rm G}{\rm {\rm }}^{{\rm app}}_{\rm m}\sim 0$. The gray area corresponds to *ϕ* ≫ (*K*_3_*r*′)^−1^. In this region ${\rm G}{\rm }^{{\rm app}}_{\rm m}$ acquires the maximum value and *K*^app^ decays as the inverse of *ϕ* (**B**). Parameters for the simulation were: *K*_1_ = 0.2 μM^−1^, *K*_3_ = 1 μM^−1^, *r*′ = 100 μM and *γgk*_mL_[P_T_] = 100 μM.

Interestingly, the model allows us to define the maximum expression of the reporter (${\rm G}{\rm }^{{\rm app}{\rm }}_{\rm m}$) and the affinity constant of the device (*K*^app^) as functions of the normalized strength of the RBS receptor (*ϕ*). According to the expressions (16), (17) and (18), Figure 4 illustrates the Michaelian effect of [L] and *ϕ* in the device. Notice that ${\rm G}{\rm }_{\rm m}^{{\rm app}}$ reaches its maximum value when *ϕ* ≫ (*K*_3_*r*′)^−1^ is satisfied (gray region in the Figure 4B). Under such a condition, the affinity of the device increases, reducing the value of *K*^app^ as *ϕ*^−1^ (see Figure 4B).

In this model, ${\rm G}^{{\rm app}}_{\rm m}$ ranges from zero at *ϕ* = 0 to a maximal value, determined by the efficiency of the machinery, formally *γgk*_mL_[P_T_]. Conversely, *K*^app^ at *ϕ* = 0 acquires its maximum value $K_1^{-1}$, corresponding with ${\rm G}^{{\rm app}}_{\rm m}=0$.

It is worth mentioning that extreme cases address an ideal trend and they would not match real behavior. This is due to considerations of the simple model, such as the availability of ligand and resources, as well as the extreme variation of the *ϕ* values, which are far to be satisfied under physiological conditions. Unlike Hill approximation, this simple equation allows us to see in a qualitative way the role of this device according to the variation of its components.

It is worth to note the similarity of equation (18) with the so-called reversible acompetitive inhibition in enzymology (8). Input affinity and signal amplitude is affected in a saturable way by the RBS strength as it occurs with the inhibitor in the enzymological model. However, in our case such a dependence occurs in an activatory fashion.

### Mathematical model including leakiness predicts the effect of receptor on output basality

The model presented in this section is a generalization of the previous one. In contrast to the simplified version, this model incorporates two lactone-independent expression pathways: one mediated by the free receptor and another by the naked promoter. Departing from the chemical reactions previously presented in Equations (4), (5) and (6), we now add the following binding equilibrium:

(19) \begin{eqnarray*} {{\rm R}}_2 + {{\rm P}} \rightleftarrows {{\rm S}}_{{\rm R}} &\Longrightarrow & {K}_2 = [{{\rm S}}_{\rm R}]/([{{\rm R}}_2][{{\rm P}}]). \end{eqnarray*}

The concentration of the protein reporter [G] is determined by the equation based on the intermediates species—including the free promoter activity mediated by *k*_m0_—as described in the following ODEs:

(20) \begin{eqnarray*} &{{\rm d}{\rm }[{\rm m}{\rm G}] \over {\rm d}t}=& k_{{\rm m0}} [{\rm P}] + k_{{\rm mR}}[{\rm S}_{\rm R}] + k_{{\rm mL}}[{\rm S}_{\rm L}] -k_{{\rm {\rm m}d}}[{\rm m}{\rm G}] \end{eqnarray*}

(21) \begin{eqnarray*} &{{\rm d}[{\rm G}{\rm }] \over {\rm d}t} = & k_{{\rm p}}[{\rm m}{\rm G}] -k_{{\rm pd}}[{\rm G}]. \end{eqnarray*}

Analogously to the simple model, we shall write an expression of [G*] based on equilibrium constants and total concentration of the chemical species. As detailed in the mathematical appendix, the resulting expression in its compact version is:

(22) \begin{eqnarray*} [{\rm G}^*]&=& a_1 \left(\frac{a_2 + {\rm L}}{a_3 + {\rm L}}\right), \end{eqnarray*}

where *a*_1_, *a*_2_ and *a*_3_ are functions of *ϕ* (detailed in Equations (53), (54) and (55) of the mathematical appendix), accounting for amplitude signal, basality and ligand affinity, respectively.

By considering the two limit cases [L] = 0 and [L] = ∞ of the (22) and defining the turning point as that [L] = L_0.5_ for G_m_/2, we connect mathematical parameters with measurable values of the transfer function as follows:

(23) \begin{eqnarray*} &{\rm G}_0 =& \frac{a_1 a_2}{a_3} = \gamma g {\rm P}_{\rm T} \frac{k_{{\rm m0}}+ k_{{\rm mR}}K_2r^{\prime }\phi }{1+K_2r^{\prime }\phi } \end{eqnarray*}

(24) \begin{eqnarray*} &{\rm G}_{\rm m} =& a_1 =\gamma g {\rm P}{\rm }_{\rm T} \frac{ k_{{\rm m}0} + k_{{\rm mL}{\rm }}K_3r^{\prime }\phi }{1 +K_3r^{\prime }\phi } \end{eqnarray*}

(25) \begin{eqnarray*} &{\rm L}{\rm }_{0.5} =& a_3-2a_2. \end{eqnarray*}

**Figure 5.**
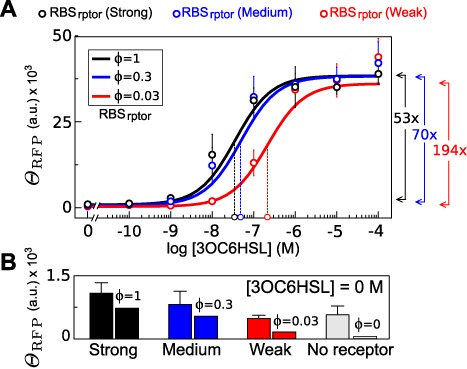
Transfer function (**A**) and basal expression (**B**) obtained from fitting our mathematical model including leakiness with real data. It explains the effect of receptor in amplitude signal and ligand affinity. Arrows show the times of variation from the ratio of G_m_/G_0_ from fitted model equation. Model was manually fitted to data using the following parameter values: *γk*_m0_[P_T_] = 85 M, *K*_2_*r*′ = 5, *K*_3_*r*′ = 500, *ε*_R_ = 10, *ε*_L_ = 10 and $K_1^{-1}=3\times 10^{-6}$ M. Pearson test was applied to the different constructs giving the following correlation coefficients: 0.996, 0.987 and 0.987 for weak, medium and strong, respectively. Comparison of model and real data leakiness in the panel (B) gave a correlation coefficient of 0.984.

The parameters G_0_, G_m_ and L_0.5_ correspond to the basal expression, maximal expression and turning point, respectively. Continuous lines in Figure 5A show that the effect of *ϕ* is similar to the observed one from the simple model behavior for signal amplitude (G_m_) and turning point (L_0.5_) (see Figure 4A for comparison). Leakiness by free receptor gives rise to a basal signal at [L] = 0 that also depends on *ϕ* in a saturable way, according to Equation (23).

This expression, although less treatable than the simpler version, offers a predictable behavior useful for data fitting. Figure 5 shows the fitting of experimental data using values of normalized strength for the strong (*ϕ* = 1), medium (*ϕ* = 0.3) and weak (*ϕ* = 0.03) RBS parts, as are described in the parts registry collection (see Materials and Methods). The fitting using the Equation (22) gave a basal output and turning point that fairly matched the experimental values (see Table 1). In the same way, amplitudes from the model and the experiments followed a similar trend as suggested by Pearson correlation between data and the model, as detailed in Figure 5.

## DISCUSSION

Despite the potential applicability of Synthetic Biology in biomedicine and environmental issues, a proper characterization of global behavior of devices in a reliable and predictable way is missing. In this regard, the use of biological meaningful models allows us to determine the behavior of tunable genetic devices.

Inspired by well established formal approaches from enzymology, our results show that the impact of tunable parts such as the RBS receptor, the plasmid copy number (which would modulate [P_T_]) and even the RBS directly affecting RPF, can be predictably studied without the use of the Hill function approach. From an engineering perspective, the model reveals that the sensation by an inducible device can be adjusted by varying the receptor levels. A strategy based on the changes of the receptor expression is worth considering rather than complicated protein engineering.

Interestingly, the model offers a suitable explanation about the lack of cooperativity observed in the experimental results: only one molecule of lactone is required for the activation complex. This is in agreement with evidence found in literature for Lux and homologous quorum sensing receptors. The free ligand receptor formation allows us to explain the leakiness mediated by free receptor described in this work. Such evidence provides some concerns about the behavior of the engineered Lux device, specially when positive cooperativity is desired (13,15).

Noteworthy, for the RBS_rptor_ (*strong*) we observed a reduction in the receptor levels, despite its expression took place under a constitutive promoter. This reduction may affect the device response when scaling up the system. These experimental results may suggest the existence of an indirect negative interaction between inducible gene expression, in our case RFP, and other nonregulated genes, such as LuxR. We speculate that the metabolic load associated to device induction could be responsible for this behavior. Looking at Figure 5B, the higher levels of basal RFP expression in constructs with and without LuxR in absence of lactone can be interpreted in similar terms. This latter example illustrates the possibility that experimental setup may not adjust well to our model premises, being the nonorthogonality of genetic devices another possible source of discrepancies. The predictive value of the model precisely relies on the assumption that its premises are fulfilled, and therefore deviations from the expected behavior could be a hint that some premises are broken. Further research incorporating factors such as metabolic load and crosstalks might improve the predictive capabilities of the model.

Bearing all this in mind, principles of organization described in this work may offer an evolutionary insight, in particular, in processes of adaptation or even the emergence of some type of diseases. According to our results, mutations at the level of receptor expression may offer a finer tuning process than those occurring at the polypeptidic chain of the receptor. In this context, the work provides a proof of concept for an interesting evolutionary perspective of the principles of biological design extracted from a synthetic biology approximation.

## CONCLUSION

The use of an enzymology-based approach provides a framework for the study and reliable characterization of synthetic devices uncovering interesting connections of the principles of organization of natural systems. Further work on the extension of an enzymological approach to the study of more complex genetic behaviors would be of great interest for the controllability and development of new synthetic genetic devices.

## MATHEMATICAL APPENDIX

According to the chemical reactions described in Equations (4), (5), (19) and (6), the expression of the protein reporter [G] (which can be tuned by the modification of the RBS parts of the genetic device) is determined by two parts: one constitutive, related to the receptor expression [R_T_] and another one inducible, affected by the presence of ligand [L].

### Derivation of the constitutive expression of the receptor in relation to the relative RBS strength

We assume that the receptor expression is controlled by a constitutive promoter with a linear degradation of the gene product. Prefixes m and p are used for transcription and translation of related species and kinetic constants, respectively. The resulting equations for the receptor are split in its transcription and translation processes as follows:

(26) \begin{equation*} {{\rm d}[{\rm m}{\rm R}_{\rm T}] \over {\rm d}{\rm }t}=k^{\prime }_{{\rm m0}} - k^{\prime }_{{\rm md}} [{\rm m}{\rm R}_{\rm T}] \end{equation*}

where [mR_T_] is the mRNA species that, at the steady state, is defined as $[{\rm mR}_{\rm {\rm T}}^*]=k^{\prime }_{{\rm {\rm m0}}}/k^{\prime }_{{\rm {\rm md}{\rm }}}$. The translation process is described by the following ODE and the respective steady state for the value of total receptor concentration:

(27) \begin{eqnarray*} &{{\rm d}[{\rm R}_{\rm T}] \over {\rm d}t}=&k^{\prime }_{\rm p} [{\rm mR}_{\rm T}]-k^{\prime }_{{\rm pd}} [{\rm R}_{\rm T}] \end{eqnarray*}

(28) \begin{eqnarray*} &[{\rm R}{\rm }_{\rm T}^*]=&k^{\prime }_{\rm p} k^{\prime }_{{\rm m0}}/(k^{\prime }_{{\rm md}} k^{\prime }_{{\rm pd}}). \end{eqnarray*}

Normalizing $k^{\prime }_{\rm p}$ by the kinetic constant of the canonical RBS sequence B0034 (cita web) denoted by $k_{\rm p}^{\rm o}$ we get that $k^{\prime }_{\rm p}= \phi k_{\rm p}^{\rm o}$. In this way we define *ϕ* as the relative strength of a particular RBS part, respecting the canonical sequence. Grouping all the constants except *ϕ* we obtain

(29) \begin{equation*} r= k_{\rm p}^{\rm o} k^{\prime }_{{\rm m0}}/(k^{\prime }_{{\rm md}} k^{\prime }_{{\rm pd}}). \end{equation*}

Then, we can express $[{\rm R}{\rm }_{\rm T}^*]$ as a function of *ϕ* as follows

(30) \begin{equation*} [{\rm R}_{\rm T}^*]=\phi r. \end{equation*}

### Derivation of the inducible part

According to the chemical reactions described in Equations (4), (5), (6) and (19), the expression of the protein reporter [G], is determined by the following equation:

(31) \begin{equation*} {{\rm d}{\rm }[{\rm G}{\rm {\rm }}] \over {\rm d}{\rm }t}= k_{\rm 0} [{\rm P}{\rm }] + k_{\rm R} [{\rm S}_{\rm R}] + k_{{\rm L}} [{\rm S}{\rm }_{{\rm L}}] - k_{\rm d} [{\rm G}{\rm }]. \end{equation*}

In this equation, we capture by order of the summations: (i) the basal expression due to the naked promoter [P], (ii) the expression by the promoter bound to receptor without ligand [S_R_], (iii) the promoter bound ligand-receptor complex, denoted as [S_L_] and (iv) the natural degradation of the protein [G]. Processes of transcription and translation are collapsed in the following kinetic constants for the alternative types of G synthesis: *k*_0_, *k*_R_ and *k*_L_. This is possible by the steady-state assumption also for the RNA species. Transcription and translation processes are defined by these two ODEs:

(32) \begin{eqnarray*} &{{\rm d}[{\rm m}{\rm G}{\rm }] \over {\rm d}t}=& k_{{\rm m0}} [{\rm P}{\rm }] + k_{{\rm mR}}[{\rm S}_{\rm R}] + k_{{\rm mL}}[{\rm S}_{\rm L}] -k_{{\rm md}}[{\rm m}{\rm }{\rm G}] \end{eqnarray*}

(33) \begin{eqnarray*} &{{\rm d}{\rm }[{\rm G}{\rm {\rm }}] \over {\rm d}t} =& k_{{\rm p}}[{\rm m}{\rm G}] -k_{{\rm pd}}[{\rm G}{\rm }]. \end{eqnarray*}

Giving rise to the value of the protein G at the steady state:

(34) \begin{equation*} [{\rm G}{\rm }^*]= k_{{\rm p}}/(k_{{\rm md}} k_{{\rm pd}}\left(k_{{\rm m0}} [{\rm P}] + k_{{\rm mR}{\rm }} [{\rm S}{\rm }_{\rm R}] + k_{{\rm mL}} [{\rm S}_{\rm L}]\right)) \end{equation*}

We now normalize *k*_p_ by $k_{\rm p}^{\rm o}$, obtaining a relative strength for the RBS of the inducible part, defined as

(35) \begin{equation*} \gamma =k_{\rm p}/k_{\rm p}^{\rm o}. \end{equation*}

By grouping some kinetic constants we get

(36) \begin{equation*} g=k_{\rm p}^{\rm o}/(k_{{\rm md}} k_{{\rm pd}}) \end{equation*}

obtaining the following expression:

(37) \begin{equation*} {\rm G}^*= \gamma g \left(k_{{\rm m0}} [{\rm P}{\rm {\rm }}] + k_{{\rm mR}} [{\rm S}{\rm }_{\rm {\rm R}}] + k_{{\rm mL}} [{\rm S}_{{\rm L}}]\right) \end{equation*}

where *γ* represents the normalized RBS strength used in a particular construct. Now we shall proceed to get an expression in terms of parameters of the system. For the sake of simplicity, we will start with obtaining the expression as a function of *R*_2_ and then to give the final expression in terms of the RBS strengths. This provides a more tractable and intuitive expression for the behavior of the gene expression. According to model premises, [R_T_] and [P_T_] satisfy the following conservation expressions:

(38) \begin{eqnarray*} &[{\rm R}_{\rm T}] =& [{\rm R}{\rm }] + [{\rm R}{\rm }_{\rm 2}] + [{\rm Q}] + [{\rm S}_{\rm R}] +[{\rm S}_{\rm L}] \end{eqnarray*}

(39) \begin{eqnarray*} &[{\rm P}{\rm }_{\rm T}] =& [{\rm P}{\rm }] + [{\rm S}_{\rm R}] + [{\rm S}_{\rm L}]. \end{eqnarray*}

[R_T_] ≫ [P_T_], [S_R_] and [S_L_] are not considered in Equation (38) giving rise to a simpler equation:

(40) \begin{eqnarray*} &[{\rm R}_{\rm T}] =& [{\rm R}] + [{\rm R}_{\rm 2}] + [{\rm Q}]. \end{eqnarray*}

Taking the equilibrium constants of Equations (4), (5), (19) and (6), we express [S_R_], [S_L_] and [Q] in function of [R_2T_] and [L] and [P]:

(41) \begin{eqnarray*} &[{\rm Q}] =& K_{\rm 1}{\rm R}_{\rm 2}[{\rm L}] \end{eqnarray*}

(42) \begin{eqnarray*} & [{\rm S}_{\rm R}] =& K_{\rm 2}[{\rm P}][{\rm R}{\rm }_{\rm 2}] \end{eqnarray*}

(43) \begin{eqnarray*} &[{\rm S}{\rm }_{\rm L}] =& K_{\rm 3}K_{\rm 1}[{\rm P}{\rm }][{\rm R}_{\rm 2}][{\rm L}{\rm }]. \end{eqnarray*}

Now, from the equation conservation defined in Equation (40), we replace the [Q] expression of the Equation (41) and we get

(44) \begin{equation*} [{\rm R}_{\rm 2}]={[{\rm R}_{{\rm 2T}}] \over (1+K_{\rm 1}[{\rm L}])}. \end{equation*}

Now, from the conservation equation of [P_T_] (see Equation (11)) we derive the expression of [P] by replacing [S_R_] and [S_L_] from Equations (42) and (43). Accordingly, we obtain

(45) \begin{equation*} [{\rm P}{\rm }]={[{\rm P}_{\rm T}] \over \left( 1+ (K_{\rm 2}+K_{\rm 1} K_{\rm 3} [{\rm L}])[{\rm R}{\rm }_{\rm 2}]\right)} \end{equation*}

Replacing [Q], [S_R_] and [S_L_] from Equations (41), (42) and (43), and [P] from (45) in Equation (37), we obtain after reordering

(46) \begin{equation*} [{\rm G}{\rm }^*]= \gamma g {\rm P}_{\rm T}\frac{k_{{\rm m0}} + \left(k_{{\rm mR}{\rm }} K_2 + k_{{\rm mL}} K_{\rm 1}K_{\rm 3} [{\rm L}]\right)[{\rm R}{\rm }_{\rm 2}{\rm }] }{1 + (K_{\rm 2} + K_{\rm 1}K_{\rm 3} [{\rm L}])[{\rm R}_{\rm 2}{\rm }]}. \end{equation*}

Now, replacing [R_2_] by the eq. (44) we obtain

(47) \begin{equation*} {\rm G}{\rm {\rm }}^*={\gamma g [{\rm P}{\rm }_{\rm T}] (\sigma _{\rm 1} +\sigma _{\rm 2}{\rm } {\rm L}) \over \sigma _{\rm 3} +\sigma _{\rm 4}{\rm L}} \end{equation*}

where

(48) \begin{eqnarray*} \sigma _1=\frac{ k_{{\rm m0}}}{{\rm R}_{{\rm 2T}}} + k_{{\rm mR}}K_{\rm 2}{\rm } ,\;& \sigma _2 =\frac{ k_{{\rm m0}}K_{\rm 1}{\rm }}{{\rm R}{\rm }_{{\rm 2T}}} +k_{{\rm mL}}K_{\rm 1}K_{\rm 3} \end{eqnarray*}

(49) \begin{eqnarray*} \sigma _3=\frac{1}{{\rm R}_{{\rm 2T}}} +K_{\rm 2} ,\;& \sigma _{\rm 4}=\frac{K_{\rm 1}}{{\rm R}{\rm {\rm }}_{{\rm 2T}}} +K_{\rm 1}K_{\rm 3}. \end{eqnarray*}

We can rewrite Equation (47) as follows

(50) \begin{equation*} {\rm G}{\rm {\rm }}^*=\gamma g {\rm P}{\rm }_{\rm T} \frac{\sigma _2}{\sigma _4} \left(\frac{\sigma _1/\sigma _2 + {\rm L}{\rm }}{\sigma _3/\sigma _4 + {\rm L}}\right) \end{equation*}

and we finally obtain the expression (18) by defining

(51) \begin{eqnarray*} a_1=\gamma g {\rm P}_{\rm T} \sigma _2/\sigma _4&\; a_2=\sigma _1/\sigma _2&\; a_3=\sigma _3/\sigma _4. \end{eqnarray*}

### Relation between [R_2T_] and [R_T_]

For the [R_2T_] value, the simplification allows to establish a constant relation with [R_T_] just solving the second degree equation: 4*K*_0_[R_2T_]^2^ + (1 − 4*K*_0_[R_T_])[R_2T_] + *K*_0_[R_T_]^2^ = 0. Taking the positive solution of the equation and assuming that *K*_0_ ≫ 1, we obtain

(52) \begin{equation*} [{\rm R}_{2{\rm T}}] \approx {[{\rm R}_{\rm T}] \over 2}= {\phi r \over 2} = \phi r^{\prime }. \end{equation*}

For the general case, rewriting (51) applying (52) we obtain the expressions

(53) \begin{eqnarray*} a_1&=&\gamma g [{\rm P}_{\rm T}] (k_{{\rm m}0} + k_{{\rm mL}}K_3r^{\prime }\phi )/(1 +K_3r^{\prime }\phi ) \end{eqnarray*}

(54) \begin{eqnarray*} a_2&=&K_1^{-1}\left(\frac{ k_{{\rm m}0}+k_{{\rm mR}} K_2r^{\prime }\phi }{ k_{{\rm m}0} + k_{{\rm mL}}K_3r^{\prime } \phi }\right) \end{eqnarray*}

(55) \begin{eqnarray*} a_3&=&K_1^{-1} (1+ K_2r^{\prime }\phi )/(1 + K_3r^{\prime }\phi )). \end{eqnarray*}

Simplified model (15) can be similarly treated applying (52) to obtain (18).

## References

[1] Endy D. (2005). Foundations for engineering biology. Nature.

[2] Tyson J.J., Chen K.C., Novak B. (2003). Sniffers, buzzers, toggles and blinkers: dynamics of regulatory and signaling pathways in the cell. Curr. Opin. Cell Biol..

[3] Baldwin G., Bayer T., Dickinson R., Ellis T.P.S., Freemont R.I.K., Polizzi K., Stan G.B. (2012). *Synthetic Biology: A Primer*.

[4] Mutalik V.K., Guimaraes J.C., Cambray G., Mai Q.A., Christoffersen M.J., Martin L., Yu A., Lam C., Rodriguez C., Bennett G. (2013). Quantitative estimation of activity and quality for collections of functional genetic elements. Nat. Methods.

[5] Kwok R. (2010). Five hard truths for synthetic biology. Nature.

[6] Mukherji S., van Oudenaarden A. (2009). Synthetic biology: understanding biological design from synthetic circuits. Nat. Rev. Genet..

[7] Wang B., Kitney R.I., Joly N., Buck M. (2011). Engineering modular and orthogonal genetic logic gates for robust digital-like synthetic biology. Nat. Commun..

[8] Cornish-Bowden A. (2004). *Fundamentals of Enzyme Kinetics*.

[9] Trunnell N., Poon A., Kim S., Ferrell J.J. (2011). Ultrasensitivity in the regulation of Cdc25C by Cdk1.. Mol. Cell.

[10] Gardner T.S., Cantor C.R., Collins J.J. (2000). Construction of a genetic toggle switch in Escherichia coli. Nature.

[11] Hooshangi S., Thiberge S., Weiss R. (2005). Ultrasensitivity and noise propagation in a synthetic transcriptional cascade. Proc. Natl Acad. Sci. U.S.A..

[12] Huang C.Y., Ferrell J. Jr (1996). Ultrasensitivity in the mitogen-activated protein kinase cascade. Proc. Natl Acad. Sci. U.S.A..

[13] Weber M., Buceta J. (2013). Dynamics of the quorum sensing switch: stochastic and non-stationary effects. BMC Syst. Biol..

[14] Goutelle S., Maurin M., Rougier F., Barbaut X., Bourguignon L., Ducher M., Maire P. (2008). The Hill equation: a review of its capabilities in pharmacological modelling. Fundam. Clin. Pharmacol..

[15] Garcia-Ojalvo J., Elowitz M.B., Strogatz S.H. (2004). Modeling a synthetic multicellular clock: repressilators coupled by quorum sensing. Proc. Natl Acad. Sci. U.S.A..

[16] Balagadde F.K., Song H., Ozaki J., Collins C.H., Barnet M., Arnold F.H., Quake S.R., You L. (2008). A synthetic Escherichia coli predator-prey ecosystem. Mol. Syst. Biol..

[17] Swinburne I.A., Miguez D.G., Landgraf D., Silver P.A. (2008). Intron length increases oscillatory periods of gene expression in animal cells. Genes Dev..

[18] Chappell J., Jensen K., Freemont P.S. (2013). Validation of an entirely in vitro approach for rapid prototyping of DNA regulatory elements for synthetic biology. Nucleic Acids Res..

[19] Aron U. (2006). *An Introduction to Systems Biology: Design Principles of Biological Circuits*.

[20] Michaelis L., Menten M.L. (1913). Die Kinetik der Invertinwirkung. Biochem. Z..

[21] Williams J.W., Cui X., Levchenko A., Stevens A.M. (2008). Robust and sensitive control of a quorum-sensing circuit by two interlocked feedback loops. Mol. Syst. Biol..

[22] Tu K.C., Long T., Svenningsen S.L., Wingreen N.S., Bassler B.L. (2010). Negative feedback loops involving small regulatory RNAs precisely control the Vibrio harveyi quorum-sensing response. Mol. Cell.

[23] Zucca S., Pasotti L., Mazzini G., De Angelis M.G.C., Magni P. (2012). Characterization of an inducible promoter in different DNA copy number conditions. BMC Bioinformatics.

[24] Fuqua C., Winans S.C., Greenberg E.P. (1996). Census and consensus in bacterial ecosystems: the LuxR-LuxI family of quorum-sensing transcriptional regulators. Annu. Rev. Microbiol..

[25] Welch M., Todd D.E., Whitehead N.A., McGowan S.J., Bycroft B.W., Salmond G.P. (2000). N-acyl homoserine lactone binding to the CarR receptor determines quorum-sensing specificity in Erwinia. EMBO J..

[26] Urbanowski M.L., Lostroh C.P., Greenberg E.P. (2004). Reversible acyl-homoserine lactone binding to purified Vibrio fischeri LuxR protein. J. Bacteriol..

[27] van Kessel J.C., Ulrich L.E., Zhulin I.B., Bassler B.L. (2013). Analysis of activator and repressor functions reveals the requirements for transcriptional control by LuxR, the master regulator of quorum sensing in Vibrio harveyi. MBio.

[28] Salis H.M., Mirsky E.A., Voigt C.A. (2009). Automated design of synthetic ribosome binding sites to control protein expression. Nat. Biotechnol..

[29] Kelly J.R., Rubin A.J., Davis J.H., Ajo-Franklin C.M., Cumbers J., Czar M.J., de Mora K., Glieberman A.L., Monie D.D., Endy D. (2009). Measuring the activity of BioBrick promoters using an in vivo reference standard. J. Biol. Eng..

[30] Saeidi N., Wong C.K., Lo T.M., Nguyen H.X., Ling H., Leong S.S., Poh C.L., Chang M.W. (2011). Engineering microbes to sense and eradicate Pseudomonas aeruginosa, a human pathogen. Mol. Syst. Biol..

